# TLR4 Activation Promotes Podocyte Injury and Interstitial Fibrosis in Diabetic Nephropathy

**DOI:** 10.1371/journal.pone.0097985

**Published:** 2014-05-19

**Authors:** Jin Ma, Steven J. Chadban, Cathy Y. Zhao, Xiaochen Chen, Tony Kwan, Usha Panchapakesan, Carol A. Pollock, Huiling Wu

**Affiliations:** 1 Renal Medicine and Transplant Research Group, Royal Prince Alfred Hospital, Sydney, New South Wales, Australia; 2 Renal Research Group, Kolling Institute of Medical Research, Royal North Shore Hospital, Sydney, New South Wales, Australia; 3 Sydney Medical School, The University of Sydney, Sydney, New South Wales, Australia; University of Washington, United States of America

## Abstract

Toll like receptor (TLR) 4 has been reported to promote inflammation in diabetic nephropathy. However the role of TLR4 in the complicated pathophysiology of diabetic nephropathy is not understood. In this study, we report elevated expression of TLR4, its endogenous ligands and downstream cytokines, chemokines and fibrogenic genes in diabetic nephropathy in WT mice with streptozotocin (STZ) diabetes. Subsequently, we demonstrated that TLR4^−/−^ mice were protected against the development of diabetic nephropathy, exhibiting less albuminuria, inflammation, glomerular hypertrophy and hypercellularity, podocyte and tubular injury as compared to diabetic wild-type controls. Marked reductions in interstitial collagen deposition, myofibroblast activation (α-SMA) and expression of fibrogenic genes (TGF-β and fibronectin) were also evident in TLR4 deficient mice. Consistent with our *in vivo* results, high glucose directly promoted TLR4 activation in podocytes and tubular epithelial cells *in vitro*, resulting in NF-κB activation and consequent inflammatory and fibrogenic responses. Our data indicate that TLR4 activation may promote inflammation, podocyte and tubular epithelial cell injury and interstitial fibrosis, suggesting TLR4 is a potential therapeutic target for diabetic nephropathy.

## Introduction

Diabetic nephropathy (DN) is the leading cause of chronic kidney disease globally and continues to grow in incidence and prevalence in parallel with the pandemic of type 2 diabetes [1]. Between 20–40% of those with diabetes develop progressive nephropathy leading to stage 5 chronic kidney disease. The pathogenesis of DN remains incompletely understood and consequently specific therapies are lacking. Although historically considered as a largely ‘non-inflammatory’ disease process, emerging evidence from experimental and clinical studies has indicated that inflammatory processes facilitated by innate immune responses play a significant role in the development and progression of DN [2], [3].

TLRs are innate immune receptors expressed by immune cells, including macrophages, dendritic cells, lymphocytes, and non-immune cells including kidney tubular epithelial cells (TECs), endothelial cells and podocytes [4].TLRs recognize pathogen-associated molecular patterns present on microorganisms but also recognize endogenous ligands released by damaged or stressed tissues including heat-shock proteins (HSPs), high-mobility group box 1 (HMGB1), fibronectin and biglycan [5]–[7]. Upon activation TLRs signal via an adaptor molecule MyD88, leading to translocation of nuclear factor kappa B (NF-κB) with consequent upregulation of pro-inflammatory cytokines and chemokines, in turn initiating local inflammation and leukocyte accumulation. These pro-inflammatory cytokines and chemokines are known to participate in the pathogenesis of DN [2].

TLR2 and TLR4 are actively involved in the development of kidney diseases in a sterile environment. TLR4 and/or 2 are required for the development of kidney damage in response to ischemia-reperfusion injury [8], [9], cisplatin-induced nephrotoxicity [10] and glomerulonephritis [11], [12]. Increased expression of TLR2 and 4 has been found in monocytes from patients with type 1 and 2 diabetes mellitus [13], [14], suggesting TLR2 and 4 may play an important role in the inflammatory milieu which characterizes diabetes. Recent experimental studies support this concept as TLR2 or TLR4 deficiency attenuated the pro-inflammatory state generated in wild-type mice with STZ-induced diabetes [15]–[19]. Blockade of TLR4 signalling also showed reno-protective effects in type 2 diabetic mice [20].

TLRs are expressed by intrinsic kidney cells which are known to be important in the pathogenesis of DN. TECs are an important source of cytokines in kidney disease [8], [21]. TLR4 signalling triggers TEC production of CCL2 and consequent macrophage recruitment in ischemia-reperfusion injury [8]. As TEC TLR4 upregulation has been reported in human DN, correlating with both macrophage accumulation and loss of kidney function, a role for TLR4-induced inflammation in DN appears likely [15]. Podocytes play important roles in the pathogenesis of proteinuria in DN. Given recent findings that podocyte TLR4 expression was upregulated in membranoproliferative glomerulonephritis and appeared to contribute to glomerular injury by modulating expression of pro-inflammatory chemokines [22], it is plausible that TLR activation is a mediator of DN.

Advanced human DN is marked histologically by progressive glomerulosclerosis and accumulation of myofibroblasts and extracellular matrix in the glomeruli and tubulo-interstitium. Emerging evidence has indicated that TLRs may provide a link between inflammation and fibrosis in chronic injury. TLR4 modulated liver fibrosis via a TGF-β-dependent manner in three different models of hepatic disease, suggesting that TLR4 may function as a molecular link between pro-inflammatory and pro-fibrogenic signals in liver [23]. Whether TLRs provide a link between inflammation and fibrosis in DN remains unknown.

Given the importance of TLR signalling in various models of kidney diseases and the likely role of innate immunity in modulating inflammatory processes in the development of DN, we examined the impact of TLR4 deficiency on both the development of DN *in vivo* and on kidney cell responses to glucose *in vitro*. We herein present data which demonstrate how the absence of TLR4 signalling attenuated podocyte and TEC inflammatory responses to high glucose and protected mice from early and late injury in STZ-induced DN.

## Subjects and Methods

### Animals

Wild-type (WT) Balb/c mice were obtained from the Animal Resource Centre (Perth, Australia). TLR4-deficient mice on a Balb/c background were provided by Animal Service of Australian National University with permission from Professor S Akira (Osaka University, Osaka,Japan). The mice were housed in a specific pathogen-free facility at the University of Sydney. Male mice aged 8–9 weeks were used in all experiments. All animal experiments were performed with the approval of the animal ethics committee of the University of Sydney.

### Induction of diabetes

Male WT and TLR4^−/−^ mice were fasted for 4 hours then injected intraperitoneally with 55 mg/kg STZ (Sigma-Aldrich) or vehicle for 5 consecutive days. Mice with a blood glucose level over 16 mmol/L were considered to be diabetic. Animals were sacrificed at week 6 (WT n = 12; TLR4^−/−^ n = 9), week 12 (WT n = 10; TLR4^−/−^ n = 13) and week 24 (WT n = 12; TLR4^−/−^ n = 12). The control mice were 5 per group.

### Sample harvest

Urine was collected over 16 hours on the day prior to sacrifice. Blood and kidney tissues were harvested at sacrifice. Tissue slices were fixed with 10% neutral-buffered formalin for paraffin embedding, frozen in OCT compound (Sakura Finetek Inc., Torrance, CA) or snap frozen in liquid nitrogen for mRNA extraction.

### Quantification of albuminuria and urine creatinine

Urine albumin was quantified using the Mouse Albumin ELISA Quantitation Set according to the manufacturer's instructions (Bethyl Laboratories, Montgomery, TX, USA). Briefly, plates (BD Biosciences) were coated with a goat anti-mouse albumin antibody, then rinsed and blocked with assay diluent. Diluted urine samples were applied in triplicate to the plate, along with a reference serum albumin standard dilution series, and incubated for 1 hour. The plate was rinsed and incubated with HRP-conjugated mouse albumin antibody for 1 hour. Once washed, the plate was incubated with substrate solution for 10 minutes and then with stop solution. Urine albumin concentration was analyzed by microplate reader software (BMG Labtech).

Urine creatinine was measured enzymatically by the Biochemistry Department of Royal Prince Alfred Hospital, Sydney, Australia.

### Real-time RT-PCR

Total RNA was extracted using TRIzol (Invitrogen). cDNA was synthesized using oligo(dT)16 (Applied Biosystems, Foster City, CA) and the SuperScript III reverse transcriptase kit (Invitrogen) according to the manufacturer's instructions. cDNA was amplified in Universal Master Mix (Applied Biosystems) with gene-specific primers and probes, using the Rotor-Gene 6000 (Corbett Life Science). Specific TaqMan primers and probes for IL-6, TNF-α, CCL2, CXCL10, HSP70, biglycan, HMGB1 and GAPDH were previously described [8]. TaqMan primers and probes for TGF-β1 (Mm01178820_m1), fibronectin (Mm01328142_m1) and KIM-1 (Mm00506686_m1) were obtained from Applied Biosystems. All of the results are expressed as ratio to GAPDH.

### Histology

Periodic acid–Schiff (PAS) and Picro-Sirius red (PSR) staining were performed on 3 µm formalin-fixed kidney sections. Glomerular tuft area (A_G_) was measured by microscopy using DP2-BSW software V2.2, OLYMPUS. Mean glomerular volume (V_G_) was calculated using the formula described by Weibel and Gomez [24]; *V_G_ = (β/k)×(A_G_)^3/2^*, where k = 1.1 (size distribution coefficient) and β = 1.38 (shape coefficient for spheres). In each glomerular tuft, mesangial area was defined as positive staining with PAS and enumerated by image analysis software (Image Pro Premier 9.0), expressed as percentage of total glomerular area [25]. Total glomerular cellularity was determined by tallying nuclei in glomerular cross-section using ImageJ. Interstitial collagen on PSR-stained sections was assessed by point counting using an ocular grid as described by McWhinnie *et al* [26] in at least 20 consecutive fields (×400 magnification). Only interstitial collagen was counted, and vessels and glomeruli were excluded. The result was expressed as the percentage of positive staining point per field.

### Immunohistochemistry

Staining for CD68 and F4/80 was performed on acetone-fixed frozen sections (7 µm) and endogenous biotin was blocked using a biotin blocker system (DAKO, Carpinteria, CA). For TLR4 and WT1 detection, formalin-fixed sections were deparaffinized and boiled in 10 mM sodium citrate buffer (pH 6.0). Sections were then incubated with 10% normal horse serum followed by 60-minute incubation with primary antibodies: a rat anti-mouse CD68 antibody (ABD Serotec Inc., Oxford, UK), a rat anti-mouse F4/80 (ABD Serotec Inc.), rabbit anti-mouse-TLR4 antibody (Invitrogen, Catalogue No. 48-2300) for glomerular [22], goat anti-mouse-TLR4 antibody (Santa Cruz Biotechnology, Catalogue No sc-12511) for tubular [8], rabbit anti-WT1 antibody (Abcam, Cambridge, UK), or concentration-matched IgG as an isotype negative control. The sections were exposed to H_2_O_2_ and then incubated with biotinylated anti-rat IgG or anti-rabbit IgG (BD Biosciences, Pharmingen), or anti-goat IgG (Vector Laboratories Inc). A Vector stain ABC kit (Vector Laboratories Inc) was applied to the tissue followed by DAB solution (DAKO). Immunostaining for α-SMA was performed on formalin-fixed paraffin sections using Dako ARK Peroxidase for Mouse Primary Antibodies (DAKO) according to the manufacturer's instructions.

### Immunofluorescence

Podocin staining was performed on 7 µm acetone-fixed frozen sections. After blocking with 10% normal horse serum, sections were incubated with a rabbit anti-NPHS2 antibody (Abcam.) at 4°Covernight. For detection, sections were incubated with an Alexa Fluor 488-conjugated anti-rabbit antibody for 1 hour.

### Quantification of immunostaining

α-SMA immunostaining was assessed using ImageJ in the periglomerular area (between Bowman's capsule and surrounding tubules) around glomerular cross-sections (×400 magnification) and expressed as the percentage of staining around the perimeter of Bowman's capsule as described [27]. Glomerular F4/80^+^ cells were counted in glomerular cross-sections (×400 magnification). Analysis of interstitial CD68^+^ cells was performed by assessing twenty consecutive high-power fields (HPFs; ×400 magnification) of the cortex in each section. Using an ocular grid, the number of cells staining positively for each antibody was counted and expressed as cells per field. The glomerular area expressing podocin was assessed in glomerular cross-sections using ImageJ and expressed as the percentage of positive staining area of glomerulus [28]. Mean number (N) of WT1^+^ or CD68^+^ cells was estimated from a single histologic section using a method described by Venkatareddy *et al.* [29]. The apparent mean nuclear caliper diameter (d) of WT1^+^ or CD68^+^ cells was measured using image analysis software (DP2-BSW V2.2, Olympus) on ×400 magnification sections with an Olympus BX60 Microscope. The true mean nuclear caliper diameter (D) of WT1^+^ or CD68^+^ cells was then calculated using the quadratic formula *D = (d-T+((d-T)^2^+4fdT)^1/2^)/2f*, where T is the section thickness (5 µm) and f the shape coefficient (0.72). A correction factor CF from each experimental group was calculated using the equation *CF = 1/(D/T+1)*. The density (Den) of WT1^+^ or CD68^+^ cells was obtained using the equation *Den = (N_t_×CF)/(T×A_G_)*, where N*_t_* is the observed WT1^+^ or CD68^+^ cell count per tuft area. Finally, N_−_ was determined using the formula *N = Den×V_G_*.

### Primary culture of TECs and podocytes

Mouse primary TECs were isolated and cultured according to the method as described previously [8]. The isolation of mouse glomeruli was performed using the Dynabeads perfusion method [30] with modifications. The kidneys were perfused with 10^7^ Dynabeads and the cortex was cut into small pieces (1–2 mm^3^) and digested in 2 mg/mL collagenase at 37°C for 30 minutes. The collagenase-digested tissue was pressed through a 100 µm sieve and centrifuged at 200×*g*. The pellet was resuspended and glomeruli-containing Dynabeads were gathered in a magnetic field. The glomeruli were pipetted onto a 40 µm nylon sieve to remove free Dynabeads and collected by washing through an inverted nylon sieve.

Isolated glomeruli were seeded on collagen-coated culture dishes (BD Biosciences) in the DMEM/F-12 medium containing 5% fetal bovine serum supplemented with 0.5% ITSS, 100 U/mL penicillin and 100 mg/mL streptomycin (Invitrogen) and incubated at 37°C. The experiments were commenced after the cells had reached about 80% confluence.

### High glucose stimulation of podocytes or TEC *in vitro*

Cultured podocytes or TECs at 80% confluence were rinsed and incubated with serum-free DMEM/F12 medium with 0.5% ITSS supplement for podocytes or serum free K1 medium for TECs for 48 hours. The cells were exposed to 30 mM D-glucose (Invitrogen) or mannitol (5.5 mM glucose+24.5 mM mannitol) as osmotic controls in fresh 0.5% ITSS-supplemented DMEM/F12 medium for podocytes or K1 medium for TECs for 12 hours. After stimulation, the cells were harvested for mRNA or nuclear protein extraction.

### Electrophoretic mobility shift assay (EMSA)

NF-κB DNA binding activity was measured by EMSA as described previously [31]. Nuclear extracts from podocytes were prepared using a NucBuste Protein Extraction Kit (Novagen, Darmstadt, Germany) and EMSA was performed using the DIG Gel Shift Kit (Roche Applied Science, Indianapolis, IN) as per the manufacturer's instructions. The results were analyzed using ImageJ software.

### Statistical analysis

All data are presented as mean ± SD or mean ± SEM. The differences between two groups were analyzed by *t* tests, and multiple groups were compared using one- or two-way ANOVA with *post-hoc* Bonferroni's correction (Graph Pad Prism 6.0 software, San Diego, CA). *p* values less than 0.05 were considered statistically significant.

## Results

### WT and TLR4^−/−^ mice developed equivalent STZ-induced diabetes

**Figure 1:**
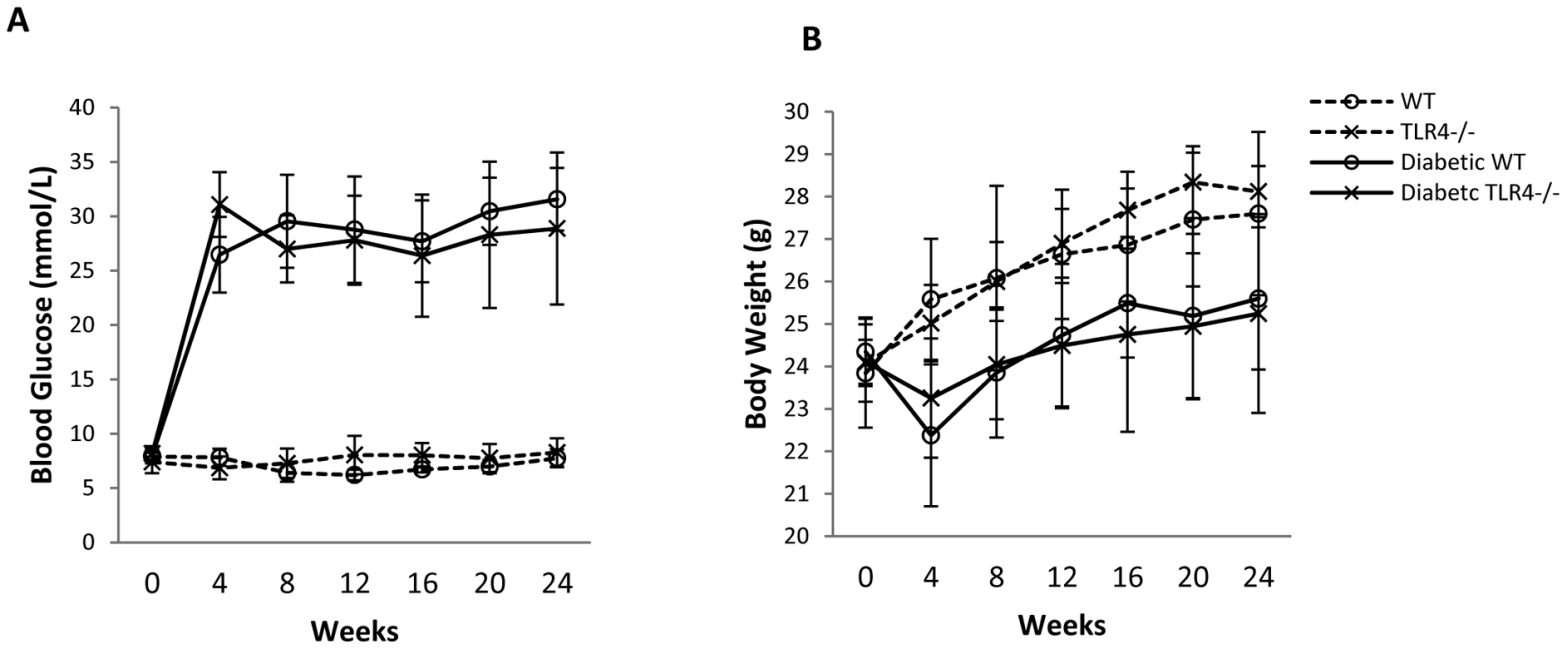
WT and TLR4^−/−^ mice treated with streptozotocin display a similar profile of hyperglycemia (A) and changes in body weight (B) over a 24 week period including week 6 (WT n = 12; TLR4^−/−^ n = 9), week 12 (WT n = 10; TLR4^−/−^ n = 13), week 24 (WT n = 12; TLR4^−/−^ n = 12) for diabetes groups and non-diabetes controls were 5 mice per group. The data are present as the means ± SD.

WT and TLR4^−/−^ mice treated with STZ displayed a similar profile in the progression of hyperglycemia (Figure 1A) and changes of body weight (Figure 1B) over a 24 week period.

### Expression of TLR4 and its endogenous ligands were upregulated in early DN

**Figure 2:**
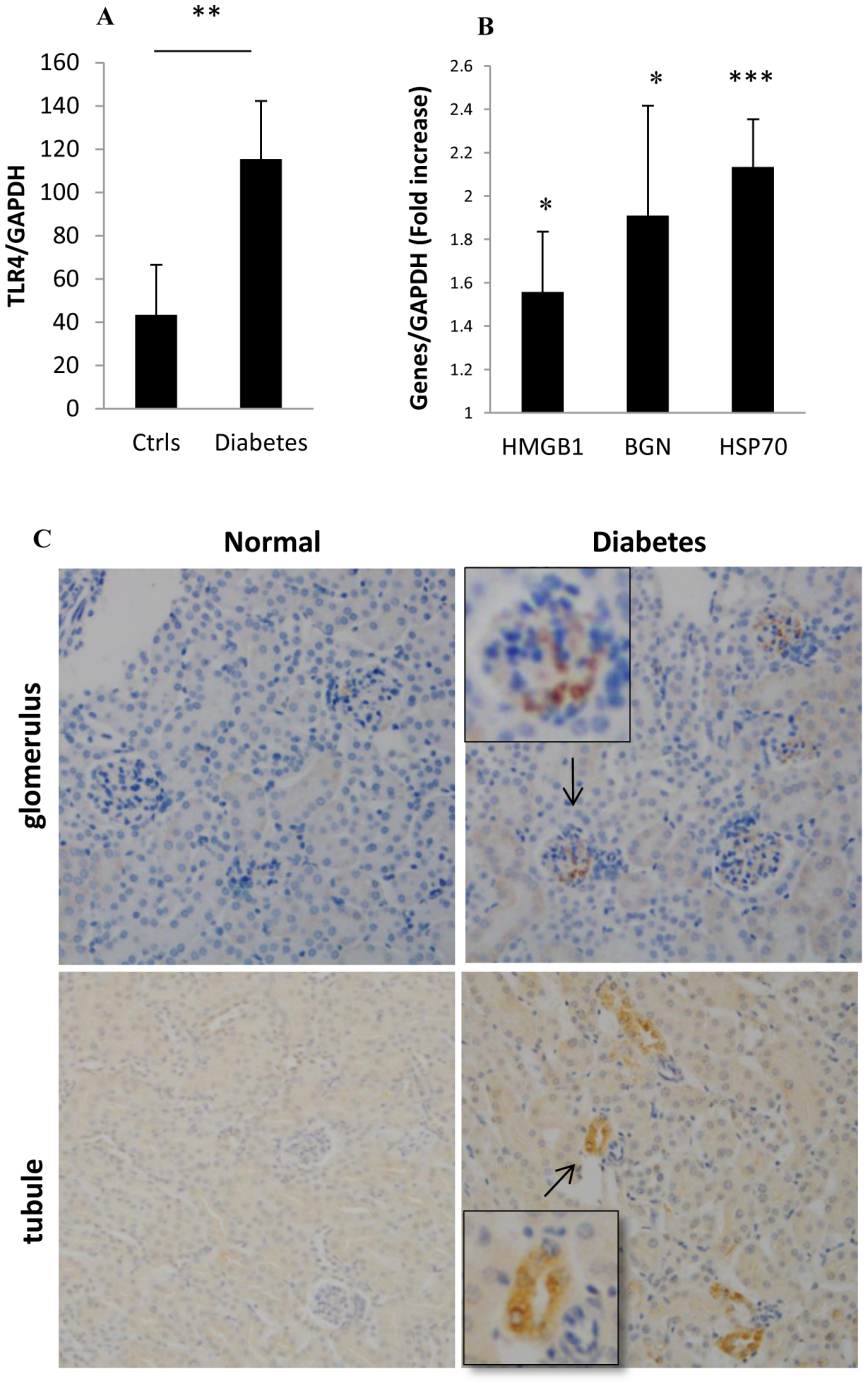
Expression of TLR4 and its endogenous ligands was upregulated in the early diabetic kidney in WT mice. mRNA expression of TLR4 and its ligands in kidney was upregulated at 10 weeks after diabetes induction (A & B). The data are presented as means ± SD; * *p*<0.05; ** *p*<0.01; *** *p*<0.001. n = 7 per group. (C) Representative sections of kidney were stained with a rabbit anti-mouse-TLR4 polyclonal antibody in the top panels and a goat anti-mouse-TLR4 polyclonal antibody in the bottom panels.

TLR4 mRNA expression in whole kidney was elevated at 10 weeks in WT mice with DN (WT-DN) compared to controls (Figure 2A). Immunohistochemistry staining indicated upregulation of TLR4 protein in tubules and glomeruli consistent with a podocyte distributionin WT-DN (Figure 2C). mRNA expression of endogenous ligands including HSP70, HMGB1 and biglycan were up-regulated in WT-DN (Figure 2B).

### TLR4 deficiency attenuated albuminuria

**Figure 3:**
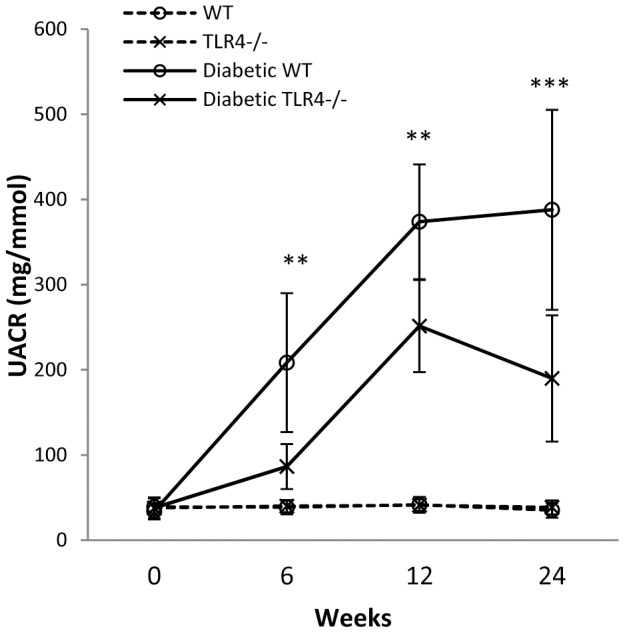
TLR4 deficiency attenuated albuminuria in DN compared to WT mice at week 6, 12, and 24. The data are present as the means ± SD; ** *p*<0.01; *** *p*<0.001. The number of animals per group was described in Figure 1.

After STZ injection, WT diabetic mice developed significant albuminuria from week 6 compared to non-diabetic controls (albumin∶creatinine ratio (UACR) 208.5±25.8 vs 34.1±9.2 mg/mmol). Albuminuria plateaued between weeks 12–24, at approximately 10-fold higher values than WT non-diabetic controls (Figure 3). Albuminuria was halved in TLR4^−/−^ mice with DN compared to WT-DN at all time points (Figure 3).

### TLR4 deficiency reduced kidney hypertrophy and glomerular injury

**Figure 4:**
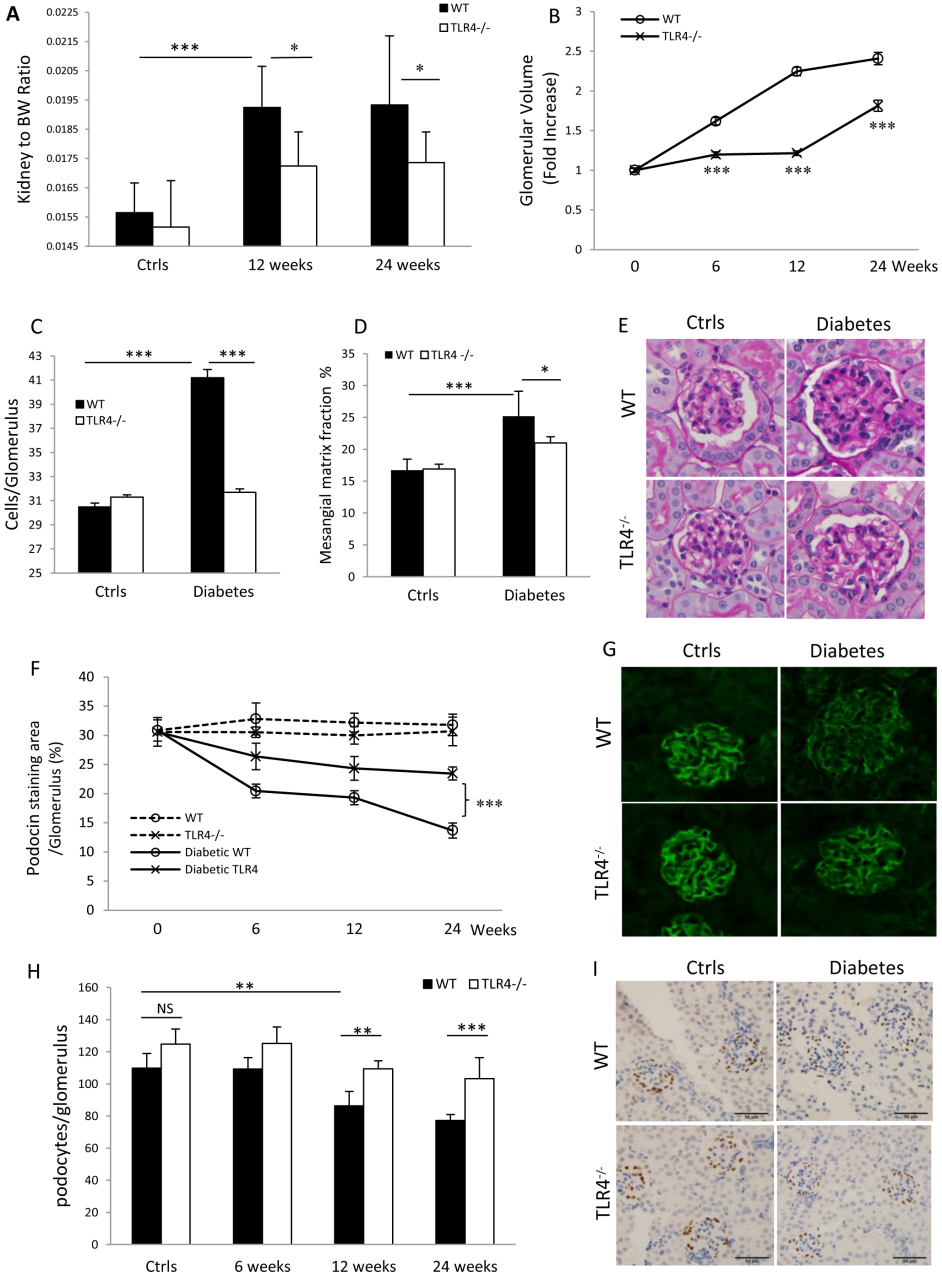
TLR4 deficiency diminished glomerular injury in DN. WT diabetic mice showed significant glomerular injury including increased kidney to body weight ratio (A), glomerular volume (B), glomerular cellularity (C, week 24), glomerular mesangial matrix (D & E), decreased podocin staining (F) and WT1^+^ podocyte numbers (H & I) compared to non-diabetic WT controls. These were attenuated in TLR4^−/−^ diabetic mice. (E) Photomicrographs of representative sections of glomerular changes at 24 weeks on PAS staining (×400 magnification). (G) Representative sections of glomeruli were stained for podocin at week 24, with similar staining intensity evident in non-diabetic WT and TLR4^−/−^ mice but reduced staining in diabetic mice with a substantially greater reduction seen in WT versus TLR4^−/−^ diabetic mice. (I) Representative sections were stained for WT1 at week 24. The data are presented as the means ± SEM; * *p*<0.05; *** *p*<0.001; *** *p*<0.0001. The number of animals per group was described in Figure 1.

Kidney hypertrophy is one of the early histological manifestations of DN [32]. WT-DN mice developed significant kidney hypertrophy as indicated by a 23% increase in kidney to body weight ratio compared to non-diabetic WT controls (Figure 4A). This was attenuated in TLR4^−/−^ diabetic mice. WT-DN kidneys displayed a progressive increase in glomerular volume, 1.6-fold increase at week 6, 2.3-fold at week 12 and 2.4-fold at week 24 compared to non-diabetic WT mice (Figure 4B & E, *p*<0.0001). In contrast, TLR4^−/−^ diabetic kidneys showed no significant glomerular hypertrophy at week 6 or 12 (Figure 4B & E) compared to TLR4^−/−^ non-diabetic controls and a significant reduction in glomerular volume compared to WT-DN at week 24 (Figure 4B & E). Glomerular hypercellularity was observed in WT-DN at week 24 with a 30% increase in total glomerular cellularity compared to non-diabetic controls (Figure 4C & E, *p*<0.0001). TLR4^−/−^ diabetic kidneys displayed no increase in glomerular cellularity (Figure 4C & E). Mesangial matrix expansion was evident on comparison of PAS-stained glomerular cross sections from DN-WT animals as compared to WT and TLR4^−/−^ non-diabetic kidneys, but was less apparent in kidneys from TLR4^−/−^ diabetic mice (Figure 4E). This impression was confirmed by computerized morphometric analysis of PAS-stained sections which revealed significant mesangial expansion in WT-DN which was significantly reduced in TLR4^−/−^ diabetic kidneys compared to WT-DN at week 24 (Figure 4D).

Albuminuria is accompanied by podocyte damage, loss, or reorganization of podocyte-associated molecules [33].To evaluate podocyte injury, immunofluorescent staining was performed for podocin. A progressive reduction in podocin staining was observed in WT-DN, significant at all time points compared to non-diabetic controls. TLR4^−/−^ diabetic mice also displayed reduced podocin staining, but this was significantly less pronounced than in WT-DN at week 24 (*p*<0.001, Figure 4F & G). WT1^+^ podocytes were significantly reduced at week 12 and 24 compared to non-diabetic controls but this reduction was significantly attenuated in TLR4^−/−^ diabetic mice versus WT-DN at week 24 (Figure 4H & I).

### TLR4 deficiency protected diabetic kidneys from fibrosis and tubular injury

**Figure 5:**
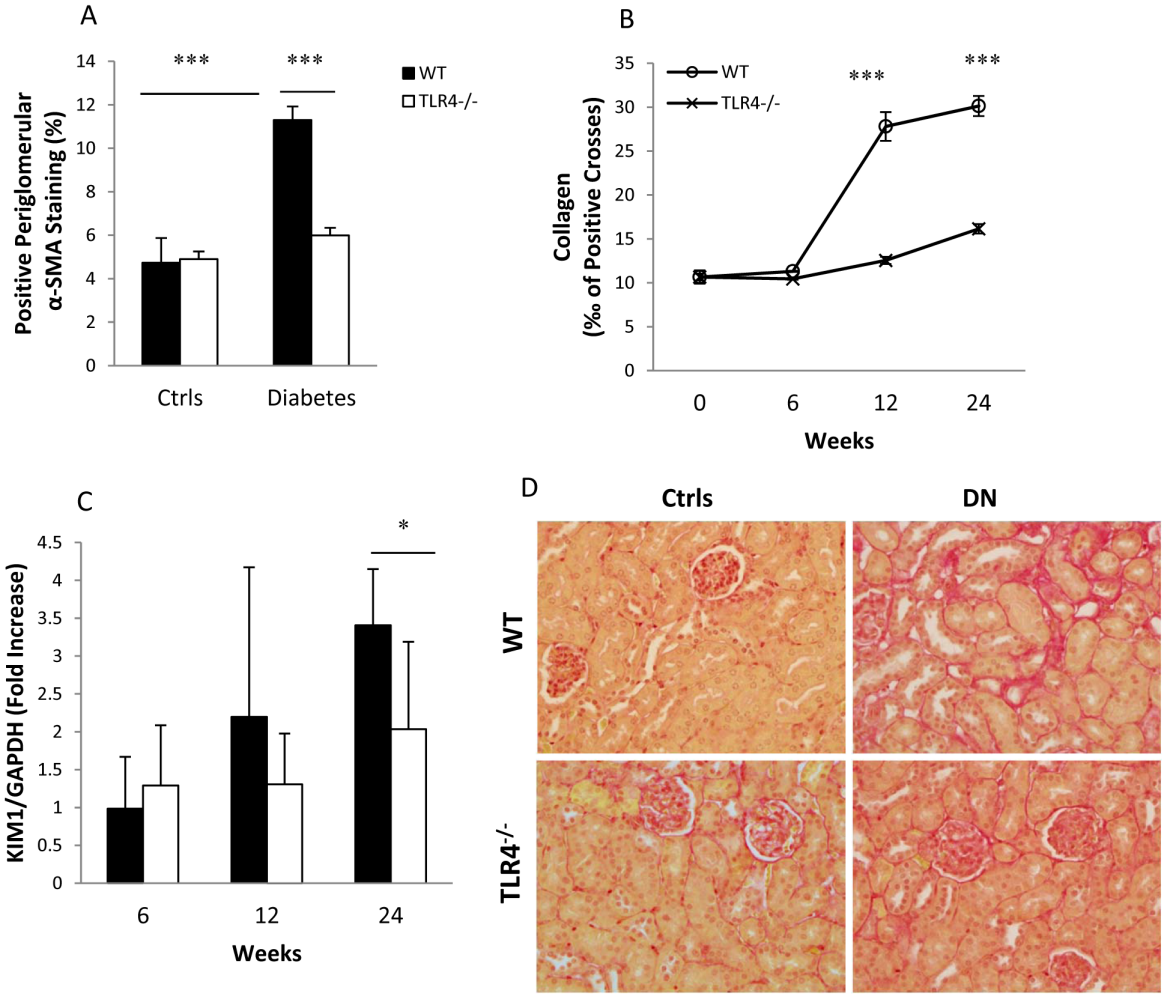
TLR4 deficiency protected diabetic kidneys from interstitial fibrosis and tubular injury. (A) Significant interstitial collagen accumulation was evident in WT but not TLR4^−/−^ mice with diabetes at 12 and 24 weeks. (B) Illustration of representative sections of the histological changes on PSR staining for collagen. (C) Immunohistochemical staining showed upregulation of α-SMA in WT, but not TLR4^−/−^ mice, with diabetes as compared to non-diabetic controls at week 24. (D) Significant upregulation of KIM-1 was apparent in WT mice with diabetes, but attenuated in TLR4^−/−^ diabetic kidneys. The data are present as the means ± SEM; * *p*<0.05; ** *p*<0.01; *** *p*<0.001. The number of animals per group was described in Figure 1.

Interstitial fibrosis is characterized by the production of interstitial matrix components such as collagen and the activation of α-SMA. A 2.7 to 3-fold increase in interstitial collagen staining was evident in WT-DN at 12 and 24 weeks versus WT controls, though no increase was observed in TLR4^−/−^ diabetic kidney (Figure 5A & B). Consistent with this, immunostaining for α-SMA indicated that periglomerular and interstitial α-SMA was significantly upregulated in WT-DN though not in TLR4^−/−^ diabetic kidneys at week 24 (Figure 5C).

Since increased expression of KIM-1 in kidneys is a marker for renal tubular injury, we examined KIM-1 mRNA expression in the kidney. Levels of KIM-1 mRNA were significantly elevated in WT-DN, but not in TLR4^−/−^ diabetic kidneys, compared to non-diabetic controls at week 24 (Figure 5D).

### CD68^+^and F4/80^+^cell accumulation was attenuated in TLR4^−/−^ diabetic kidneys

**Figure 6:**
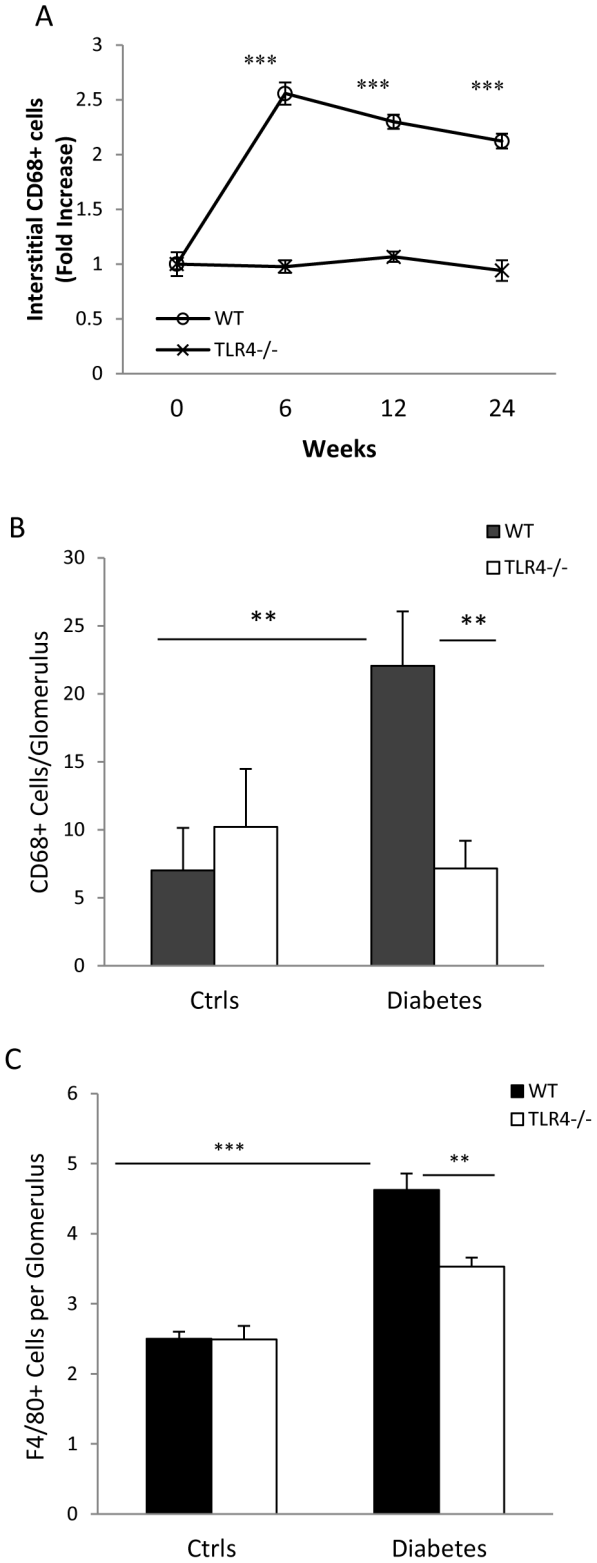
TLR4 deficiency reduced macrophage accumulation in diabetic nephropathy. Macrophage (CD68^+^ or F8/40^+^) accumulation was evident in WT mice with diabetes, but prevented by TLR4 deficiency in both the interstitial (A) and glomerular (B & C) compartments. Data are present as means ± SEM; * *p*<0.05; *** *p*<0.001.

CD68 is considered a pan-macrophage marker, predominantly expressed by tissue macrophages/monocytes. Accumulation of interstitial CD68^+^ cells was increased at least 2-fold in WT-DN at all-time points (Figure 6A), and a 3-fold increase in glomerular CD68^+^ cell accumulation was evident at 24 weeks compared with non-diabetic controls (Figure 6B). Neither interstitial nor glomerular CD68^+^ cells accumulation was seen in TLR4^−/−^ diabetic kidneys (Figure 6B). F4/80 is another marker for mouse macrophages. Consistent with the findings of glomerular CD68^+^ cell accumulation, a significant increase in glomerular F4/80^+^ cell accumulation was also observed at 24 weeks in WT-DN compared with non-diabetic controls and this was diminished in TLR4^−/−^ diabetic kidneys (Figure 6C). No significant increase of CD4^+^ and CD8^+^ T cells were found within the glomeruli and interstitium in either WT-DN or TLR4^−/−^ mice (Data not shown).

### The expression of inflammatory moleculeswas reduced in TLR4^−/−^ diabetic kidneys

**Figure 7:**
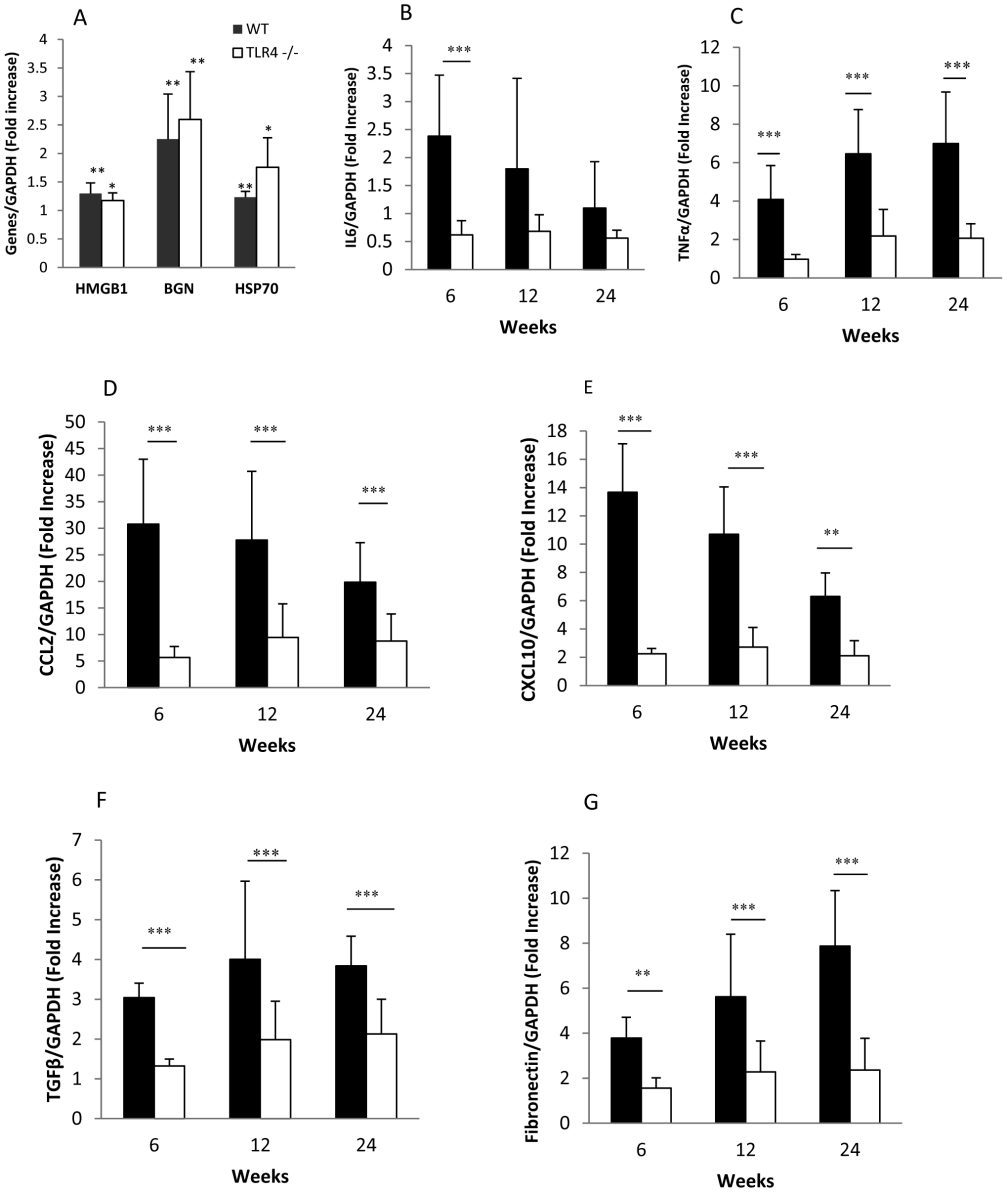
TLR4 deficiency suppressed inflammatory and fibrotic responses in diabetic kidney. The expression of endogenous TLR ligands was upregulated in WT-DN kidneys and TLR4^−/−^diabetic kidneys at week 6 compared with WT non-diabetic controls. (A). Gene expression of IL-6 (A), TNF-α (B), CCL2 (C), CXCL10 (D), TGF-β (E) and fibronectin (F) were upregulated in WT mice with diabetes, but suppressed in TLR4^−/−^ mice with diabetes, as measured by RT-PCR. Data are presented as means ± SD; ** *p*<0.01; *** *p*<0.001. The number of animals per group was described in Figure 1.

The expression of TLR endogenous ligands including HMGB1, HAS and biglycan was upregulated in WT-DN kidneys and TLR4^−/−^diabetic kidneys at week 6 post STZ injection compared with WT non-diabetic controls (Figure 7A). IL-6 mRNA expression was upregulated in WT-DN kidneys with a peak at week 6 compared with WT non-diabetic controls. This was not observed in TLR4^−/−^ diabetic kidneys (Figure 7A). TNF-α mRNA levels were progressively elevated in WT-DN from weeks 6–24 and this was significantly attenuated in TLR4^−/−^ diabetic kidneys (Figure 7B). Chemokine mRNA expression was increased in WT-DN 20–35 fold for CCL2 and 7–15 fold for CXCL10 compared with WT non-diabetic controls (*p*<0.001). This was greatly attenuated in TLR4^−/−^diabetic kidneys at all time points (Figures 7C & D). WT-DN exhibited significantly increased mRNA levels of the pro-fibrotic genes TGF-β and fibronectin compared with WT non-diabetic kidneys. Expression of both genes was suppressed in TLR4^−/−^ diabetic kidneys compared with WT-DN (Figures 7E & F).

### TLR4 mediates pro-inflammatory responses in podocytes and TECs exposed to high glucose

**Figure 8:**
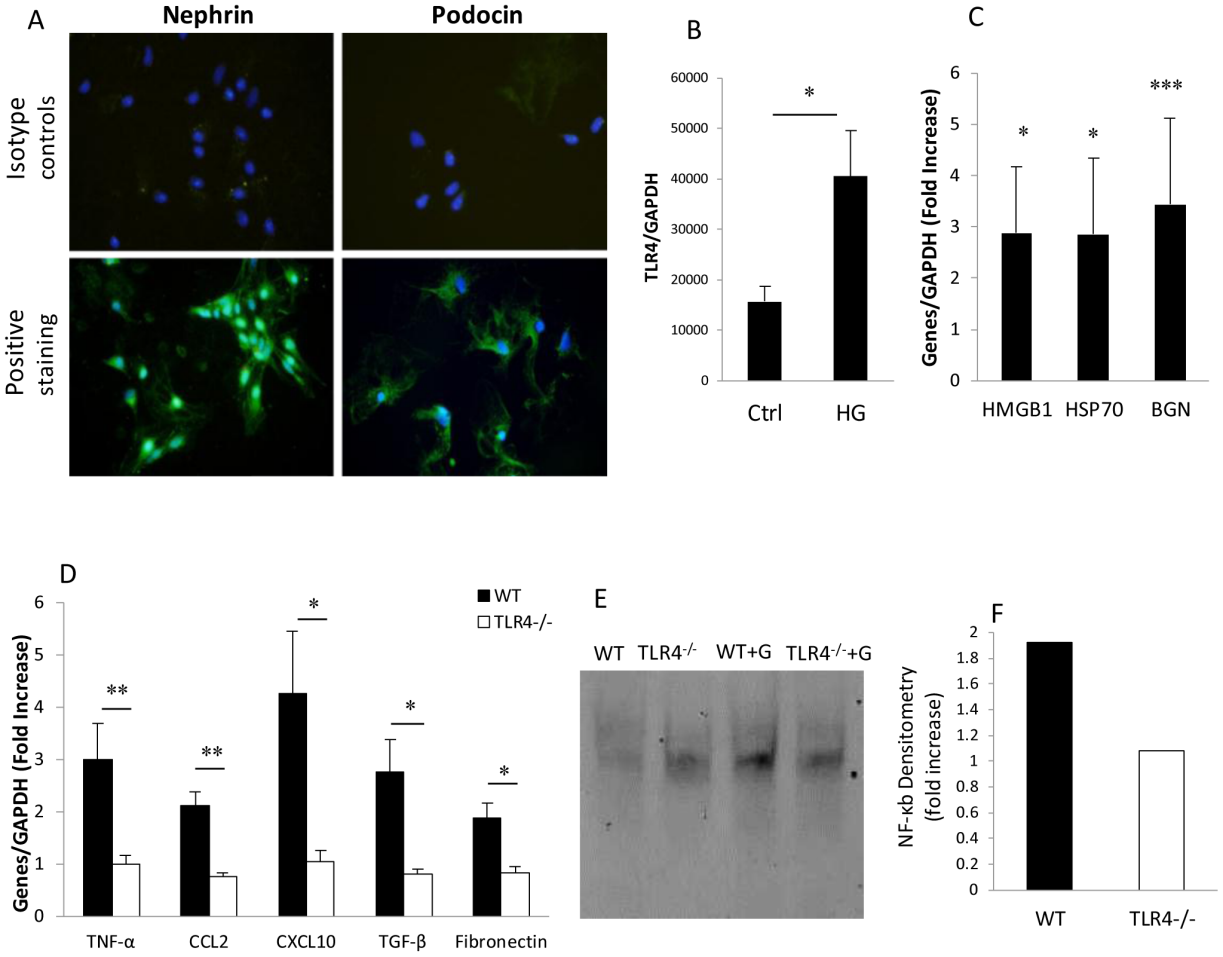
High concentration glucose induces inflammation in podocytes via TLR4. Primary podocyte cultures were defined by positive staining for the podocyte markers podocin and nephrin (A). Primary cultures of podocytes harvested from WT and TLR4^−/−^mice, were cultured in media containing physiological concentration glucose (5.5 mM glucose+24.5 mM mannitol) as an osmotic control or high-concentration glucose (5.5 mM glucose+24.5 mM glucose) for 12 hours. High glucose induced upregulation of TLR4 (B), its ligands (C) and downstream molecules (D) in WT podocytes compared to osmotic controls. Upregulation of downstream molecules induced by high glucose was not seen in TLR4^−/−^ podocytes(D). NF-κB DNA binding activity was increased by 2 fold in WT podocytes by high glucose stimulation, but not in TLR4^−/−^ podocytes (E & F). Data are presented as means ± SEM; * *p*<0.05; ** *p*<0.01; *** *p*<0.001.

To determine whether the impact of high glucose on podocytes is mediated via TLR4, primary podocyte cultures were exposed to high-concentration glucose or 5.5 mM glucose plus 24.5 mM mannitol as an osmotic control *in vitro* for 12 hours. The podocytes were confirmed by immunofluorescent staining with anti-podocin or anti-nephrin antibodies. Cells were 98% to 100% positive (Figure 8A). WT podocytes exposed to high glucose exhibited elevated mRNA levels of TLR4 and its endogenous ligands including HMGB1, HSP70 and biglycan (Figure 8B & C). mRNA expression of TLR4 downstream pro-inflammatory cytokine (TNF-α), chemokines (CCL2 & CXCL10) and pro-fibrotic genes (TGF-β & fibronectin) was increased by 2–5 fold in WT podocytes exposed to high glucose compared to the controls exposed to mannitol. These increases were diminished in TLR4^−/−^ podocytes (*p*<0.05, Figure 8D). Consistent with these results, NF-κB DNA binding activity measured by EMSA was promoted in WT podocytes but not in TLR4^−/−^ podocytes by high glucose stimulation compared with osmotic controls (Figures 8E & F).

**Figure 9:**
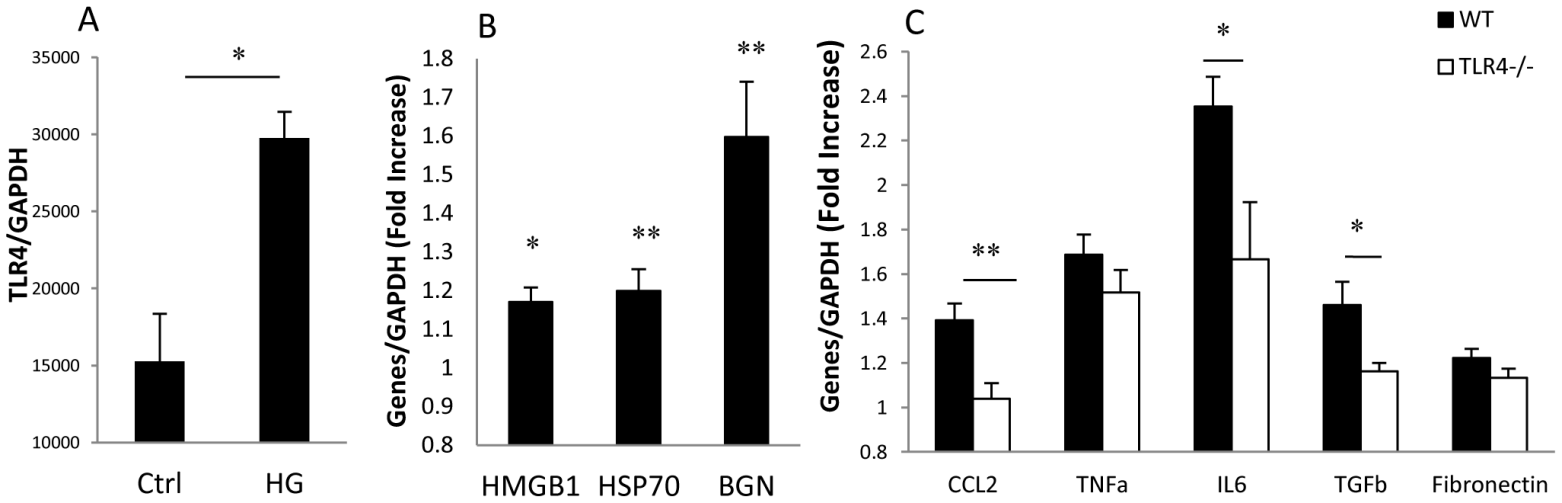
High concentration glucose induces inflammation in TECs via TLR4. Primary cultures of TECs, harvested from WT and TLR4^−/−^ mice, were cultured in media containing physiological concentration glucose (5.5 mM glucose+24.5 mM mannitol) as an osmotic control or high-concentration glucose (5.5 mM glucose+24.5 mM glucose) for 12 hours. High glucose induced upregulation of TLR4 (A), its ligands (B) and downstream molecules(C) in WT TECs compared to osmotic controls. Upregulation of downstream molecules induced by high-glucose was not seen in TLR4^−/−^ TECs (C). Data are presented as means ± SEM; * *p*<0.05; ** *p*<0.01; *** *p*<0.001.

We also observed upregulation of TLR4 and its endogenous ligands (HMGB1, HSP70, BGN) expression in primary cultured TECs exposed to 30 mM glucose (Figure 9A & B). WT-TECs exposed to 30 mM glucose expressed significantly higher levels of cytokines (IL-6 & TNF-α), chemokine (CCL2) and pro-fibrotic genes (TGF-β & fibronectin) than WT TEC osmotic controls. Up-regulation of IL-6, CCL2 and TGF-β was significantly attenuated in TLR4^−/−^ TEC cultures exposed to high glucose (Figure 9C).

## Discussion

Accumulating evidence demonstrates that inflammatory and immunologic processes play a significant role in the development and progression of DN [2], [3]. TLRs play a fundamental role in innate immune responses against invading microorganisms, but also promote sterile inflammation in a variety of diseases including atherosclerosis and diabetes. In this study, we demonstrated that the TLR4 signalling pathway is activated in WT mice with streptozotocin diabetes and that diabetic mice genetically deficient in TLR4 were protected from kidney inflammation, glomerular and tubular injury, interstitial fibrosis and albuminuria. *In vitro*, exposure to high-concentration glucose caused TLR4 pathway activation culminating in NF-κB-dependent inflammatory responses in TECs and podocytes, and these responses were attenuated in cells genetically deficient in TLR4. Taken together, our findings support earlier studies which suggested a role for TLR4 in the promotion of tubulo-interstitial inflammation in DN [15], [18] and advance this knowledge by identifying specific roles for TLR4 in mediating critical aspects of DN: inflammation, podocyte damage and kidney fibrosis.

Albuminuria is a clinical hallmark of DN, the histological correlates of which include glomerular hypertrophy, glomerular basement membrane thickening and podocyte damage including effacement of foot processes. Podocyte depletion has been identified as a strong predictor of DN progression [34]. The mechanism(s) of podocyte damage and depletion and whether podocytes contribute to glomerular inflammation in DN are not well understood. TLR4 is present on kidney parenchymal cells, including podocytes, and the importance of TLR4-mediated parenchymal cell responses triggered by endogenous ligands is well recognized in other models of kidney injury, including ischemia-reperfusion [8]. Recent studies by Banas *et al.* reported that cultured podocytes constitutively expressed TLR4 and produced chemokines in response to stimulation with LPS, and that TLR4 expression by podocytes may be critical in triggering glomerular inflammation in a model of membranoproliferative glomerulonephritis [22]. In the current study, WT mice with diabetes exhibited upregulation of TLR4 in glomeruli, together with expression of endogenous ligands and downstream inflammatory cytokines (IL-6 & TNF-α) and chemokines (CCL2 & CXCL10) along with evidence of glomerular injury, strongly supports a role for TLR4 in mediating DN. Replication of these findings for podocytes *in vitro*, activation of the TLR4 pathway in podocytes in response to high glucose, and attenuation of albuminuria, glomerular injury, podocyte depletion and expression of inflammatory cytokines and chemokines in TLR4^−/−^ mice with diabetes strongly implicates TLR4 in the mediation of podocytopathy in DN.

Late stage DN is characterized by extracellular matrix deposition and progressive fibrosis involving glomerular and tubulo-interstitial compartments. TGF-β enhances production and inhibits degradation of extracellular matrix and has been reported to be upregulated in human and experimental diabetic kidney disease, suggesting TGF-β is a key driver of this process in DN [35]. Recent studies have shown that TLR4 may modulate fibrogenic responses through the TGF-β signalling pathway. Activation of TLR4 sensitizes hepatic stellate cells toward the effects of TGF-β and thereby promotes TGF-β dependent activation and collagen production.TLR4-deficient mice were protected against hepatic fibrosis in three different models of experimental liver disease, demonstrating that TLR4 signalling is required for TGF-β-dependent hepatic fibrosis [23]. In the heart, TLR4 deficiency afforded protection against left ventricular hypertrophy and cardiac fibrosis after experimental myocardial infarction [36]. TLR4^−/−^ mice subjected to unilateral ureteral obstruction showed less renal fibrosis compared to WT counterparts [37]. In the current study, substantial upregulation of TGF-β and fibronectin gene expression was evident, along with accumulation of interstitial collagen and myofibroblasts in WT-DN. By comparison, TLR4^−/−^ mice showed significantly less upregulation of fibrosis-related genes and were protected from accumulation of interstitial collagen and myofibroblasts, suggesting a critical role for TLR4 in the promotion of kidney fibrosis in DN.

Potential cellular sources of TGF-β, fibronectin and other pro-fibrotic cytokines include kidney parenchymal cells and infiltrating inflammatory cells [38]–[40]. Macrophages are the dominant inflammatory cell present in DN, recruited by CCL2 expressed by renal tubular epithelial cells in particular [41]. We found upregulation of TLR4 in renal tubules, endogeneous TLR4 ligands (HSP70, HMGB1 and biglycan) and elevated CCL2 gene expression along with significant recruitment of interstitial macrophages in WT-DN from week 6 to 24. CCL2 expression, macrophage recruitment and fibrosis were all attenuated in TLR4^−/−^ mice with diabetes.This result is consistent with a recent study in humans where over expression of TLR4 in renal tubules correlated with monocyte and macrophage accumulation in diabetic kidneys [15]. Hyperglycemia is known to promote TLR4 expression in monocytes from patients with type 1 and 2 diabetes [13], [14]. In vitro, we also found that high glucose stimulated expression of TGF-β and fibronectinin podocytes and TECs derived from WT but not TLR4^−/−^ mice. All TLRs, except TLR3, can signal via MyD88 to initiate NF-κB-dependent inflammation. Consistent with this, we found that WT cells cultured in high glucose exhibited increased NF-κB DNA binding activity and upregulation of downstream cytokines (TNF-α), chemokines (CCL2 & CXCL10) and pro-fibrotic genes (TGF-β and fibronectin). These changes were not seen in cells derived from TLR4^−/−^ mice. Our studies were unable to determine whether macrophages, podocytes, TECs or other cells are dominant in causing fibrosis in DN, but do suggest a prominent role of TLR4 activation in the process as has been demonstrated in models of hepatic fibrosis [23], cardiac fibrosis after myocardial infarction [36] and in kidney following ureteric obstruction [37], [42].

In conclusion, our studies confirm and extend previous reports which identified TLR4 as a mediator of inflammation in DN, and also demonstrate its role in podocyte damage and kidney fibrosis. Given involvement of TLR4 pathways in these pivotal phases of diabetic nephropathy, strategies to inhibit TLR4 signalling should be explored.
